# Bone mineralization in children aged 7–10 years born after ART with frozen and fresh embryo transfer

**DOI:** 10.1093/hropen/hoaf055

**Published:** 2025-09-11

**Authors:** Elisabeth Waldemar Grønlund, Anna Sophie Lebech Kjaer, Louise Laub Asserhøj, Ikram Mizrak, Tine Dahlsgaard Clausen, Per Laub Madsen, Anja Pinborg, Rikke Beck Jensen

**Affiliations:** The Fertility Clinic, Department of Gynaecology, Fertility and Obstetrics, Copenhagen University Hospital—Rigshospitalet, Copenhagen, Denmark; The Fertility Clinic, Department of Gynaecology, Fertility and Obstetrics, Copenhagen University Hospital—Rigshospitalet, Copenhagen, Denmark; Department of Paediatrics and Adolescent Medicine, Copenhagen University Hospital—Herlev Hospital, Copenhagen, Denmark; The Fertility Clinic, Department of Gynaecology, Fertility and Obstetrics, Copenhagen University Hospital—Rigshospitalet, Copenhagen, Denmark; The Fertility Clinic, Department of Gynaecology, Fertility and Obstetrics, Copenhagen University Hospital—Rigshospitalet, Copenhagen, Denmark; Department of Cardiology, Copenhagen University Hospital—Herlev Hospital, Copenhagen, Denmark; The Obstetrics Clinic, Department of Gynaecology, Fertility and Obstetrics, Copenhagen University Hospital—Rigshospitalet, Copenhagen, Denmark; Department of Clinical Medicine, University of Copenhagen, Copenhagen, Denmark; Department of Cardiology, Copenhagen University Hospital—Herlev Hospital, Copenhagen, Denmark; Department of Clinical Medicine, University of Copenhagen, Copenhagen, Denmark; The Fertility Clinic, Department of Gynaecology, Fertility and Obstetrics, Copenhagen University Hospital—Rigshospitalet, Copenhagen, Denmark; Department of Clinical Medicine, University of Copenhagen, Copenhagen, Denmark; Department of Paediatrics and Adolescent Medicine, Copenhagen University Hospital—Herlev Hospital, Copenhagen, Denmark; Department of Clinical Medicine, University of Copenhagen, Copenhagen, Denmark

**Keywords:** ART, frozen embryo transfer, BMC, BMD, childhood growth

## Abstract

**STUDY QUESTION:**

Does height-adjusted bone mineral content (BMC) at 7–10 years of age differ between children conceived after ART with frozen embryo transfer (FET) compared to children conceived after ART with fresh embryo transfer (fresh-ET) or naturally conceived (NC) children?

**SUMMARY ANSWER:**

Children conceived after FET had an increased BMC corrected for height compared with fresh-ET and NC when adjusted for confounders, a difference primarily mediated by the higher birthweight (BW) found in children conceived after FET compared with fresh-ET and NC.

**WHAT IS KNOWN ALREADY:**

Children conceived after ART with FET have a higher BW compared to NC children, while the opposite association is known for fresh-ET. In NC children, BW is positively associated with BMC and bone mineral density (BMD), but bone health in children born after ART is scarcely explored with inconsistent results.

**STUDY DESIGN, SIZE, DURATION:**

This study was a retrospective cohort study as part of the ‘Health in Childhood following Assisted Reproductive Technology’ (HiCART) cohort, consisting of 606 singletons (292 boys), conceived after FET (n = 200), fresh-ET (n = 203), and NC (n = 203) born from November 2009 to December 2013. The children were 7–10 years old when clinically examined, and the study was conducted from January 2019 to September 2021.

**PARTICIPANTS/MATERIALS, SETTING, METHODS:**

Children were identified through the Danish Medical Birth Registry and Danish IVF Registry. These registries were also used to collect information regarding ART treatment, pregnancy, and delivery. The clinical examination involved anthropometric measurements, pubertal staging, fasting blood samples, and a whole-body dual-energy x-ray absorptiometry (DXA) scan. Both parents completed a questionnaire on background information regarding themselves and their child. The three groups were compared pairwise using univariate linear regression, and possible confounders and mediators were adjusted for using multiple linear regression analysis.

**MAIN RESULTS AND THE ROLE OF CHANCE:**

Crude values of BMC corrected for height did not differ between children born after FET, fresh-ET, or NC. When adjusted for relevant confounders, children born after FET had a statistically significant higher BMC corrected for height compared with both fresh-ET and NC. After further adjustment for BW SD score, the differences in BMC corrected for height disappeared, and no statistically significant differences in BMC corrected for height between any of the three groups were found. Factors potentially affecting bone mineralization, such as calcium, parathyroid hormone (PTH), insulin-like growth factor-1 (IGF-1), lean mass, and physical activity in childhood, did not differ between the three groups.

**LIMITATIONS, REASONS FOR CAUTION:**

All relevant confounders were adjusted for, although the cause of infertility was not available, hence a risk of residual confounding. The use of a parental questionnaire on the child’s physical activity level sets a risk of information bias.

**WIDER IMPLICATIONS OF THE FINDINGS:**

Increased BW in children conceived after FET was associated with increased BMC corrected for height at age 7–10 years when compared to children conceived after fresh-ET and NC. Longitudinal studies of pregnancies and newborns after FET are needed to explore the causes of the increase in BW and the possible effects on long-term bone health.

**STUDY FUNDING/COMPETING INTEREST(S):**

The study was funded by the Novo Nordisk Foundation (grant number: NNF18OC0034092, NFF19OC0054340) and Rigshospitalet Research Foundation. A.P. has received grants from Gedeon Richter, Ferring Pharmaceuticals, Merck, and Cryos; consulting fees from Gedeon Richter, Ferring Pharmaceuticals, Merck, Cryos, and IBSA; speaker fees from Gedeon Richter, Ferring Pharmaceuticals, Merck, and Organon; and travel grants from Gedeon Richter. The remaining authors declare no conflicts of interest.

**TRIAL REGISTRATION NUMBER:**

ClinicalTrials.gov identifier: NCT03719703.

WHAT DOES THIS MEAN FOR PATIENTS? Worldwide, the use of ART is rising, especially the use of frozen–thawed embryos during the last decade. Different ART treatments carry different advantages and risks, and the health of children born after various ART treatments is of great interest to both parents and clinicians. Children conceived after the use of frozen–thawed embryos have a tendency of a higher birthweight, while children conceived after the use of fresh embryos have a tendency of lower birthweight than average. Former studies have revealed that birthweight is positively associated with bone mineralization—a marker of bone strength and health—later in life. Therefore, we compared bone mineralization in 606 children aged 7–10 years conceived after the use of frozen–thawed embryos, fresh embryos, and natural conception, respectively. We found an increased bone mineralization in children born from frozen embryos compared with both fresh embryos and natural conception. However, this difference disappeared after considering differing birthweights, suggesting that the higher bone mineralization in children born from frozen embryos was driven by the higher birthweight in this group, not the ART treatment itself. In addition, bone mineralization in children born from fresh embryos was comparable to naturally conceived children. Thus, bone mineralization in children was comparable between all three groups, which is reassuring for current and prospective parents conceiving after ART.

## Introduction

Childhood and adolescence are critical periods for bone mineralization and development (Cooper *et al.*, 2006; Kuh *et al.*, 2014). Previous studies have suggested that environmental factors affecting fetal growth may influence bone mineralization in childhood and adolescence. The mode of conception is known to play an important role in fetal growth and may therefore affect bone development later in life (Düppe *et al.*, 1997; Sayer and Cooper, 2005; Harvey *et al.*, 2010).

ART has made great progress, and individuals born after ART constitute an increasing proportion of the world’s population due to increasing rates of infertility (De Geyter *et al.*, 2020; Smeenk *et al.*, 2023). In Denmark, the latest registry data from 2019 show that children conceived after ART make up 6.3% of the annual national births (Smeenk *et al.*, 2023). In particular, the frozen embryo transfer (FET) technique has improved significantly during the past two decades, resulting in a substantial increase in the number of FET cycles conducted (Smeenk *et al.*, 2023). This technique allows for elective single embryo transfer, thus reducing the risks associated with multiple gestations while also reducing the risk of ovarian hyperstimulation syndrome (OHSS) (Berntsen and Pinborg, 2018). However, FET is also associated with a higher birthweight (BW) and an elevated risk of being born large-for-gestational-age (Ishihara *et al.*, 2014; Berntsen and Pinborg, 2018). On the contrary, fresh embryo transfer (fresh-ET) increases the risk of preterm birth, low BW, and being born small-for-gestational-age (Elias *et al.*, 2020; Westvik-Johari *et al.*, 2021). Hence, the different ART technologies affect fetal growth and thereby the size at birth of the offspring.

Earlier studies have suggested an altered bone mineralization in offspring born after ART compared with naturally conceived (NC) offspring, but the results are inconsistent (Ceelen *et al.*, 2007; Miles *et al.*, 2007; Armangil *et al.*, 2015), and only one study compared children born after FET, fresh-ET, and NC systematically (Xia *et al.*, 2022). Studies on NC children have shown a positive association between BW and bone mineral content (BMC) in early childhood (Harvey *et al.*, 2010; Martínez-Mesa *et al.*, 2013) and in adulthood (Baird *et al.*, 2011). However, other studies have concluded that postnatal growth, especially during the first year, has a more pronounced positive association with BMC than BW (Dennison *et al.*, 2005; Beltrand *et al.*, 2008; Jensen *et al.*, 2008; Heppe *et al.*, 2014).

The aim of this study was to compare bone mineralization in children conceived after FET to children conceived after fresh-ET and NC, respectively. We hypothesized that children born after FET had a higher bone mineral content than those born after fresh-ET and NC, attributed to a higher BW.

## Materials and methods

### Study design and population

This cohort study is part of the Eastern Denmark ‘Health in Childhood following Assisted Reproductive Technology’ (HiCART) cohort. The HiCART project identified children through the Danish Medical Birth Registry and Danish IVF Registry, and the cohort consists of 606 singletons (292 boys, 314 girls) born from November 2009 to December 2013. The children were divided into three groups according to mode of conception: FET (n = 200), fresh-ET (n = 203), and NC (n = 203). The children were 7–10 years of age at the time of examination. Exclusion criteria were multiple pregnancies, siblings, oocyte donation, maternal pre-existing diabetes mellitus, gestational diabetes, as well as severe illnesses in the child as assessed by a specialized pediatrician. For further details on the recruiting process and clinical examinations from the cohort, please see previous publication by Asserhøj *et al.* (2023).

### Clinical examinations

Clinical examinations were performed at Copenhagen University Hospital—Rigshospitalet, Denmark. Investigators were blinded for mode of conception during clinical examination. The clinical examination included anthropometric measurements, a fasting blood sample, pubertal staging, and a whole-body dual-energy x-ray absorptiometry-scan (DXA).

Both parents completed a questionnaire on background information regarding themselves and their child. This questionnaire also included questions regarding the physical activity level of the child. The physical activity levels were divided into groups according to how often they participated in sports: (i) never, (ii) once per week, (iii) ≥2 times per week, and their parents’ assessment of their physical activity level compared with other children of the same age: (i) more active, (ii) neutral, and (iii) less active.

The Danish IVF Registry and the Danish Medical Birth Registry were used to collect information regarding ART treatment, pregnancy, and delivery. For further details on the collection of background information, please see the previous publication by Asserhøj *et al.* (2023).

All anthropometric measurements were taken three times, and means were used. Standing height was measured to the nearest 0.1 cm with a stadiometer, and weight was measured on a digital scale to the nearest 0.1 kg. BMI was calculated (weight/height^2^ (kg/m^2^)). To compare children of different age and sex, an SD score (SDS) was calculated for BW (Maršál *et al.*, 1996). SDSs were also calculated for height, weight, and BMI using the Lambda–Mu–Sigma method with a Danish population as reference (Tinggaard *et al.*, 2014). Pubertal staging was assessed by a clinician according to Tanner’s classification Stages 1–5 (Marshall and Tanner, 1970). Whole-body DXA scans were performed with light clothing using Lunar Prodigy (GE Healthcare, Madison, WI, USA) and analyzed using enCORE software in mode ‘Enhanced’ (Version 16, GE Healthcare). As recommended by the manufacturer, calibration was performed daily using a quality assurance block and weekly using a spine phantom. Total BMC in grams (g), bone mineral density (BMD) in g/cm^2^, and lean mass in g (defined as the difference between total weight (g) and body fat (g)) were reported. BMC measurements were size-adjusted for height. Total BMC, BMD, and lean mass referred to the total body less head (TBLH) DXA measurements.

A fasting blood sample was drawn from an antecubital vein. Analysis included calcium, parathyroid hormone (PTH), insulin-like growth factor 1 (IGF-1), and insulin-like growth factor-binding protein 3 (IGFBP-3). Serum was stored at −20°C until analysis, which was performed at Copenhagen University Hospital—Rigshospitalet, Denmark. Chemiluminescence immunoassays (Immunodiagnostics Systems [IDS]-iSYS IGF-1 and IDS-iSYS IGFBP-3 assays; IDS Ltd., Boldon, Tyne and Wear, UK) were used to analyze IGF-1 and IGFBP-3 on the IDS-iSYS Multi Discipline Automated Analyzer (IDS-iSYS; Pouilly-en-Auxois, France).

For IGF-1 and IGFBP-3, SDS were calculated using a Danish reference material (Kjaer *et al.*, 2023).

### Ethical approval

Written informed consent was obtained from all participants’ parents who had full custody of the child. Written and verbal information about the project was provided before giving consent. All procedures followed the Declaration of Helsinki. The study protocol was granted approval by the Ethics Committee of the Capital Region of Denmark (H-18000122). All personal data were handled and stored according to the General Data Protection Regulations and approved by the Danish Data Protection Agency (VD-2018-310).

### Outcomes

The primary outcome was height-adjusted BMC. Secondary outcomes were BMD, lean mass (g), concentrations of IGF-1 (SDS), IGFBP-3 (SDS), calcium and PTH, and level of physical activity. In an exploratory analysis, we investigated the correlation between BW (SDS) and BMC/height and lean mass in the entire cohort consisting of children from all three modes of conception.

### Statistical analysis

Primary and secondary outcomes were compared between children conceived after FET with children conceived after fresh-ET and NC. For basic background characteristics, a pairwise comparison was made, unless otherwise specified. Continuous variables, including DXA-scan results and hormone concentrations, were analyzed using an univariate linear regression model. Results were then adjusted for possible confounders and mediators using multiple linear regression analysis. Confounders were chosen *a priori* and included parity, maternal BMI at early pregnancy, maternal education level, smoking status, breastfeeding status, child sex, child age at examination, and pubertal status, which were entered in Model 1. BW was considered a potential mediator, and the effect was therefore examined in a separate model (Model 2). Results are reported as mean differences (SD) with 95% CI. For categorical variables, a two-sided Chi-square test was used. Results were considered significant at *P* < 0.05. All analyses were performed using R for Windows version 4.3.0 and RStudio version 2023.09.0 (The R Foundation of Statistical Computing, Vienna, Austria).

For a thorough study design description, sample size calculation, and non-participant analysis, please see the previous publication by Asserhøj *et al.* (2023).

## Results

### Participants

As previously reported (Asserhøj *et al.*, 2023), children in the HiCART cohort born after FET had a significantly higher mean BW SDS than children born after fresh-ET (mean difference: 0.42; 95% CI: (0.21; 0.62)) and NC (mean difference: 0.35; 95% CI: (0.14; 0.57)) (Table 1). Sex, gestational age (GA), childhood height, weight, and BMI SDS between FET, fresh-ET, or NC did not significantly differ (Table 1).

**Table 1.** Baseline characteristics of the 606 included children.

	**FET** ^a^	**Fresh-ET** ^a^	**NC** ^a^
**Birth characteristics**	**n = 200**	**n = 203**	**n = 203**
Sex, (n (%))			
Girls	112 (56.0)	101 (49.8)	101 (49.8)
Gestational age, days	278 (12.9)	279 (11.0)	279 (10.9)
Birthweight, g	3584 (533)	3452 (483)	3471 (498)
Birthweight, SDS	0.20 (1.09)	−0.22 (1.00)	−0.16 (1.09)
**Anthropometric characteristics at age 7–10 years**	**n = 200**	**n = 203**	**n = 203**
Age at examination, years	8.44 (0.56)	8.55 (0.51)	8.58 (0.58)
Puberty,^b^ n (%)	15 (7.5)	18 (8.9)	24 (11.8)
Height, SDS	0.25 (1.00)	0.17 (0.96)	0.07 (1.06)
Weight, SDS	0.22 (1.07)	0.10 (1.06)	0.14 (1.00)
BMI, SDS	0.16 (1.16)	0.04 (1.10)	0.20 (1.01)

The baseline characteristics shown in Table 1 have previously been published (Asserhøj *et al.*, 2023).

a Values are shown as mean (SD).

b Pubertal status was determined according to ‘Marshall and Tanner’ and puberty was for girls defined as breast Stage 2, and for boys as testicular volume >3 ml.

FET, frozen embryo transfer; fresh-ET, fresh embryo transfer; NC, naturally conceived; SDS, SD score.

At the time of examination, children born after FET, fresh-ET, and NC were 8.44 (0.56), 8.55 (0.51), and 8.58 (0.58) years of age (mean (SD)). There was no statistically significant difference in the onset of puberty across the three groups (chi-square *P* = 0.31): 7.5% of children born after FET, 8.9% of those born after fresh-ET, and 11.8% of those born after NC had entered puberty (Table 1).

### Whole-body DXA scan

DXA results for children born after FET, fresh-ET, and NC are depicted in Table 2. The crude values for BMC (g), BMC/height (g/cm), BMD (g/cm^2^), and lean mass (g) did not significantly differ between children born after FET, fresh-ET, or NC (Table 2). However, when adjusted for relevant confounders (Table 2, Model 1), children born after FET had a statistically significant higher BMC (g), BMC/height (g/cm), and BMD (g/cm^2^) compared with both fresh-ET and NC. Total lean mass (g) was also significantly higher in children born after FET compared with fresh-ET but not compared to NC (Table 2, Model 1).

**Table 2.** Whole-body dual-energy x-ray absorptiometry scan results and blood test results for the included 7–10-year-old children and comparison of mode of conception.

	**FET** ^a^	**Fresh-ET** ^a^	**NC** ^a^	**Model 1** ^d^			**Model 2** ^e^		
				**FET vs fresh-ET** ^b^	**FET vs NC** ^b^	**Fresh-ET vs NC** ^b^	**FET vs fresh-ET** ^b^	**FET vs NC** ^b^	**Fresh-ET vs NC** ^b^
**DXA**	**n = 200**	**n = 203**	**n = 203**						
BMC (g)^c^	804.40 (150.57)	788.71 (132.61)	797.67 (139.55)	32.93 (5.02; 60.85)*	29.62 (1.07; 58.16)*	−3.31 (−32.72; 26.09)	22.53 (−5.08; 50.14)	18.86 (−9.47; 47.19)	−3.67 (−32.52; 25.19)
TBLH BMC/height (g/cm)^c^	5.94 (0.86)	5.81 (0.77)	5.89 (0.79)	0.20 (0.04; 0.37)*	0.17 (0.00; 0.34)*	−0.04 (−0.21; 0.14)	0.15 (−0.01; 0.31)	0.11 (−0.05; 0.28)	−0.04 (−0.21; 0.13)
TBLH BMD (g/cm^2^)^c^	0.66 (0.06)	0.66 (0.06)	0.66 (0.06)	0.02 (0.00; 0.03)*	0.01 (0.00; 0.03)*	−0.00 (−0.01; 0.01)	0.01(−0.00; 0.02)	0.01 (−0.00; 0.02)	−0.00 (−0.01; 0.01)
Lean mass (g)	19 149.88 (3,154.66)	18 985.94 (2,793.30)	19 151.16 (2,812.48)	626.33 (60.73; 1191.93)*	555.93 (−22.50; 1134.35)	−70.40 (−666.29; 525.45)	366.74 (−185.53; 919.01)	294.23 (−272.40; 860.85)	−72.52 (−649.60; 504.56)
**Blood tests**	**n = 196**	**n = 197**	**n = 198**						
IGF-I (SDS)	0.39 (0.77)	0.33 (0.74)	0.28 (0.72)	−0.00 (−0.17; 0.17)	0.12 (−0.06; 0.29)	0.12 (−0.06; 0.30)	0.02 (−0.15; 0.19)	0.14 (−0.04; 0.31)	0.11 (−0.06; 0.29)
IGFBP-3 (SDS)	0.63 (1.12)	0.60 (1.06)	0.63 (1.00)	−0.09 (−0.33; 0.16)	−0.03 (−0.28; 0.21)	0.05 (−0.20; 0.31)	−0.05 (−0.29; 0.20)	−0.00 (−0.25; 0.25)	0.05 (−0.21; 0.30)
PTH (pmol/l)	3.90 (1.51)	4.01 (1.31)	4.16 (1.30)	−0.12 (−0.41; 0.17)	−0.35 (−0.65; −0.05)*	−0.23 (−0.54; 0.07)	−0.09 (−0.39; 0.20)	−0.32 (−0.62; −0.02)*	−0.23 (−0.54; 0.08)
Calcium (mmol/l)	2.43 (0.07)	2.42 (0.07)	2.43 (0.06)	−0.00 (−0.02; 0.01)	−0.01 (−0.02; 0.01)	−0.00 (−0.02; 0.01)	0.00 (−0.01; 0.02)	−0.00 (−0.02; 0.01)	−0.00 (−0.02; 0.01)

a Values are shown as mean (SD).

b Values are shown as mean difference (95% CI). Differences between groups are calculated as mean differences with 95% CI using univariate linear regression model.

c BMC, BMC/height, BMD and lean mass refers to the total body less head (TBLH) DXA measurements.

d Model 1: mean differences are adjusted for confounders (sex, age at examination, puberty, parity, maternal BMI at early pregnancy, smoking during pregnancy, educational level (mother), and breastfeeding) with multiple linear regression analysis.

e Model 2: mean differences adjusted for same confounders as mentioned above, *including* birth weight (SDS).

* Statistically significant. Significance level *P*-value < 0.05.

BMC, bone mineral content; BMD, bone mineral density; DXA, dual-energy x-ray absorptiometry; FET, frozen embryo transfer; fresh-ET, fresh embryo transfer; NC, naturally conceived.

After further adjustment for BW SDS, the differences in BMC and BMD were attenuated, and no statistically significant differences in BMC (g), BMC/height (g/cm), BMD (g/cm^2^), or lean mass (g) between any of the three groups were found (Table 2, Model 2).

A linear regression showed that BW (SDS) was positively correlated with total BMC/height (g/cm) (b: 0.21, 95% CI: 0.14; 0.27) and total lean mass (g) (b: 755.0, 95% CI: 531.5; 978.5) in the entire cohort.

### Endocrine parameters

IGF-1, IGFBP-3, PTH, and calcium for children born after FET, fresh-ET, and NC are also presented in Table 2. In the confounder-adjusted analyses (Table 2, Model 1), calcium (mmol/l), IGF-1 (SDS), and IGFBP-3 (SDS) levels were comparable between the groups. In both our models, PTH (pmol/l) was significantly lower for children born after FET than after NC but did not differ when comparing children born after FET with children born after fresh-ET, nor when comparing children born after fresh-ET and NC.

Further adjustment for BW SDS did not change the association between the mode of conception and any of the endocrine parameters (Table 2, Model 2).

### Physical activity level

More than 50% of the children attended sports at least twice per week (FET: n = 119 (59.5%), fresh-ET: n = 112 (55.2%), NC: n = 107 (52.7%)) (Table 3), and most parents (51.8%) assessed this as an equal amount of physical activity when comparing with children of the same age (Table 4). When comparing children born after FET with children born after fresh-ET or NC, as well as comparing children born after fresh-ET with children born after NC, the frequency of sports activities during a week was similar across the groups.

**Table 3.** Physical activity level for the included 7–10-year-old children according to how often they attended club sports during a week.

	FET, n (%)	Fresh-ET, n (%)	NC, n (%)	Total, n (%)
Never	15 (7.5)	19 (9.4)	21 (10.3)	55 (9.1)
Once per week	46 (23.0)	58 (28.6)	56 (27.6)	160 (26.4)
≥2 times per week	119 (59.5)	112 (55.2)	107 (52.7)	338 (55.8)
NA	20 (10.0)	14 (6.9)	19 (9.4)	53 (8.7)

As recorded in parent questionnaire.

FET, frozen embryo transfer; fresh-ET, fresh embryo transfer; NC, naturally conceived; NA, no available data.

**Table 4.** Physical activity level for the included 7–10-year-old children compared to children of the same age.

	FET, n (%)	Fresh-ET, n (%)	NC, n (%)	Total, n (%)
More active	62 (31.0)	80 (39.4)	62 (30.5)	204 (33.7)
Neutral	107 (53.5)	95 (46.8)	112 (55.2)	314 (51.8)
Less active	14 (7.0)	16 (7.9)	10 (4.9)	40 (6.6)
NA	17 (8.5)	12 (5.9)	19 (9.4)	48 (7.9)

As recorded in parent questionnaire.

FET, frozen embryo transfer; fresh-ET, fresh embryo transfer; NC, naturally conceived; NA, no available data.

## Discussion

This large cohort study of 606 children, 7–10 years of age, conceived after different methods of ART or NC showed an increased bone mineralization in children born after FET compared with both fresh-ET and NC when adjusted for potential confounders. However, this difference disappeared after further adjustment for BW, suggesting that these findings are mediated by the higher BW found in the children born after FET compared with fresh-ET and NC. Calcium, IGF-1, and IGFBP-3 concentrations were comparable between the groups, while PTH was lower in children born after FET as compared with children born after NC. The frequency of weekly sports activities did not differ between the three groups.

Previous studies on bone mineralization in children born after ART are limited, with few included participants and hence a high risk of Type II statistical errors. Further, only one of these studies explores differences in bone mineralization between children conceived after FET vs fresh-ET. Our results are in accordance with a previous Dutch retrospective cohort study by Ceelen *et al.* (2007) that revealed no statistically significant difference in total BMC or total BMD between pubertal ART singletons and pubertal NC singletons. In contrast to our study, they only studied DXA-scan results on pubertal children (139 ART and 143 NC) and made no distinction between FET and fresh-ET. Likewise, Miles *et al.* (2007) found no statistically significant difference in BMD SDS in prepubertal children aged 4–10 years born after fresh-ET compared with NC. However, the study was relatively small with 69 fresh-ET singletons and 71 NC singletons.

Another measurement of bone strength is the tibial speed of sound by quantitative ultrasound, where higher tibial speed of sound indicates higher bone strength (Prins *et al.*, 1998). One of these studies from Armangil *et al.* (2015) found a lower tibial speed of sound level in ART than in NC newborns. However, this study was limited by a small sample size with 37 ART singletons and 51 NC singletons, all premature and appropriate-for-gestational-age, and no distinction between FET and fresh-ET. Another study calculated a BMD Z-score from the tibial speed of sound, and comparable to our study, they found no statistically significant difference in BMD Z-score when comparing 84 ART singletons with 123 NC singletons aged 1–12 years (Xia *et al.*, 2022). However, after confounder adjustment, they found that ART singletons had a higher BMD Z-score than NC singletons, and in a subset analysis, the children born after FET had a higher BMD Z-score compared to the children born after fresh-ET.

Previous studies on NC children have shown that higher BW is associated with higher BMC and BMD (Dennison *et al.*, 2005; Baird *et al.*, 2011). Our results indicated that the higher bone mineralization in the FET group compared with fresh-ET and NC was mediated by the higher BW SDS in this group, not by the conception method *per se*. Also, we found that a higher BW SDS was correlated to a higher BMC/height in the total cohort, underlining the known positive correlation between BW and bone mineralization. The BW differences between children born after FET compared with children born after fresh-ET and NC may be caused by epigenetic changes at the early embryonic or fetal development (Filiberto *et al.*, 2011; Mani *et al.*, 2020).

IGF-1 plays an important role in the growth and development of muscle and bone mass. It contributes to the regulation of osteoblast differentiation and proliferation as well as osteoclast resorptive activity, thereby regulating bone mass (Hill *et al.*, 1995, 1997; Mohan and Baylink, 1996). IGF-1 may therefore mediate the relationship between BW and BMC and BMD. Previous studies on children have shown a positive correlation between IGF-1 and bone mass (Javaid *et al.*, 2004; Breen *et al.*, 2011). However, controversy exists, as this association disappears when correcting for bone size, suggesting IGF-1 correlates more to bone size determined by height than density (Akcakus *et al.*, 2006; Jensen *et al.*, 2008). We found equal IGF-1 levels between the three groups, which therefore had no effect on the difference in bone mineralization.

Pubertal skeletal growth spurt in height, as well as the steady skeletal growth associated with increasing age, both have a large impact on bone development. Evidence suggests an inverse relationship between timing of puberty and peak bone mass in adolescents (Gilsanz *et al.*, 2011; Bonjour and Chevalley, 2014; Elhakeem *et al.*, 2019). In the comparison of children conceived after FET to children conceived after fresh-ET, we analyzed the effect of each confounder. In this comparison, adjusting for age at the time of examination was correlated to a significantly higher BMC/height. Adjusting for children who had entered puberty also showed that puberty was significantly correlated to a higher BMC/height, although it did not have as pronounced an effect as age. In a sensitivity analysis, excluding all participants who had entered puberty, children born after FET still had a significantly higher BMC, BMC/height, and BMD compared to NC, but no difference was found when comparing FET to fresh-ET (data not shown).

Physical activity and more lean mass and fat mass in childhood and adolescence are associated with a higher bone mass (Soininen *et al.*, 2018; Al-Agha *et al.*, 2019; Yang *et al.*, 2020). In our study, the frequency of weekly sports activities did not differ between the three groups, and neither did BMI (SDS). Thus, differences in physical activity and body composition did not affect our results.

Bone mineralization is also dependent on dietary supplements of calcium and vitamin D. A regular intake of calcium and vitamin D in childhood, combined with physical activity, and especially outdoor sun-exposed activities, increases bone mineralization (Yang *et al.*, 2020). However, our study did not obtain details regarding whether the reported physical activity was an outdoor sun-exposed activity or not. Interestingly, we found a statistically significantly higher PTH in children born after FET compared to children born after NC, which differs from the finding of a higher BMC per height in the FET group compared to the NC group. No differences were found in total calcium; we had no data on ionized calcium. We believe the higher PTH found in children born after FET is due to chance. Regarding micronutrients, the Danish Health Authority recommends 10 μg of vitamin D per day from birth to age four. They also recommend 500 mg of calcium per day from 1 to 9 years of age, which in Denmark is mainly covered through the diet due to a high intake of dairy products. Due to these recommendations, differences in intake of these micronutrients between the three groups are assumed to be insignificant. However, we do acknowledge the risk of confounding for unobtained data on sun exposure and intake of micronutrients.

One of the strengths of our study is the large cohort of children included and the detailed information on the different ART treatments. All children were examined in the same setting, and each child had all examinations done on the same day, minimizing inter-rater variability, with very few missing values from the clinical examinations. The age of the children at examination was similar in the three groups, including only a few pubertal children in each group. Additionally, the extent of information, including a high response rate to the parental questionnaire regarding possible confounders, and the access to the Danish Medical Birth Registry and the National Danish IVF Registry, ensured adjustments for relevant confounders. Furthermore, our previous non-participant analysis by Asserhøj *et al.* (2023) showed high similarity of participants and non-participants and thereby a small risk of selection bias.

One of the limitations was the risk of residual confounding. Although we aimed to adjust for the most relevant confounders, we were not able to adjust for the cause of infertility. Another limitation is the risk of information bias regarding the use of a parental questionnaire on the child’s physical activity level. Using such could have led to non-differential misclassification, potentially minimizing the difference in physical activity level between the groups. Another limitation to be considered was the two-dimensional measurements of BMC by DXA, whereby the length of the bone and not the density alone affected BMC. Hence, long bones in tall children tend to overestimate BMC, and short bones in short children result in underestimated BMC. In this study, we therefore adjusted BMC for height. However, as previously reported, no statistically significant difference was found in height between the three groups in the HiCART cohort (Asserhøj *et al.* 2023).

## Conclusion

Children born after FET showed an increased BMC corrected for height and BMD compared with children born after fresh-ET and NC. This difference was mediated by the higher BW SDS found in the FET group compared with fresh-ET and NC. In addition, BMC corrected for height and BMD in children born after fresh-ET were comparable to those in NC children. Factors potentially affecting BMC or BMD, as IGF-1, lean mass, and physical activity in childhood, were similar in the three groups. These results are overall reassuring. Longitudinal studies of pregnancies and newborns after FET are needed to explore the causes of the increase in BW and the possible effects on bone mineralization. Further research on long-term bone health and the risk of fractures and osteoporosis later in life is also needed.

## Data Availability

Anonymous raw data will be shared upon reasonable request.
